# Stochastic circular persistent currents of exciton polaritons

**DOI:** 10.1038/s41598-024-63725-1

**Published:** 2024-06-05

**Authors:** J. Barrat, Roman Cherbunin, Evgeny Sedov, Ekaterina Aladinskaia, Alexey Liubomirov, Valentina Litvyak, Mikhail Petrov, Xiaoqing Zhou, Z. Hatzopoulos, Alexey Kavokin, P. G. Savvidis

**Affiliations:** 1 Key Laboratory for Quantum Materials of Zhejiang Province, Department of Physics, School of Science, Westlake University, 600 Dunyu Road, Hangzhou, 310030 Zhejiang China; 2 Institute of Natural Sciences, Westlake Institute for Advanced Study, 18 Shilongshan Road, Hangzhou, 310024 Zhejiang China; 3 Spin Optics Laboratory, St. Petersburg State University, Ulyanovskaya 1, St. Petersburg, 198504 Russia; 4 Stoletov Vladimir State University, Gorky str. 87, Vladimir, 600000 Russia; 5 FORTH-IESL, P.O. Box 1527, 71110 Heraklion, Crete Greece; 6 Abrikosov Center for Theoretical Physics, Moscow Institute of Physics and Technology, Institutskiy per. 9, Moscow Region Dolgoprudnyi, 141701 Russia; 7 Department of Materials Science and Technology, University of Crete, P.O. Box 2208, 71003 Heraklion, Crete Greece

**Keywords:** Polaritons, Polaritons, Microresonators

## Abstract

We monitor the orbital degree of freedom of exciton-polariton condensates confined within an optical trap and unveil the stochastic switching of persistent annular polariton currents under pulse-periodic excitation. Within an elliptical trap, the low-lying in energy polariton current states manifest as a two-petaled density distribution with a swirling phase. In the stochastic regime, the density distribution, averaged over multiple excitation pulses, becomes homogenized in the azimuthal direction. Meanwhile, the weighted phase, extracted from interference experiments, exhibits two compensatory jumps when varied around the center of the trap. Introducing a supplemental control optical pulse to break the reciprocity of the system enables the transition from a stochastic to a deterministic regime, allowing for controlled polariton circulation direction.

## Introduction

Recently, the orbital degree of freedom has garnered significant interest in the field of polaritonics. Exciton polaritons, which are eigenmodes of optical microcavities strongly coupled with excitons in embedded semiconductor quantum wells (QWs)^1^, form macroscopic states of exciton-polariton condensates. These condensates exhibit behavior akin to a superfluid liquid^2,3^. The flow of polaritons within the condensate state imparts nonzero orbital angular momentum (OAM) to it. Researchers have investigated polariton condensates in various trap geometries such as annular^4–9^ and pot-shaped traps^10,11^, traps of complex shapes^12,13^, as well as chains and clusters of traps^14–17^ to study and manipulate their OAM. The heightened focus on this area is motivated by the vast potential applications of the orbital degree of freedom in quantum and classical information storage and processing^18–21^, as well as in optical communications^22,23^.

Polariton vortices stand out as the most striking examples of polariton condensate states possessing nonzero OAM. The spontaneous emergence of polariton vortices, manifesting as vortex-antivortex pairs and clusters, has been the subject of thorough investigation^24–26^. Another research focus is on the deliberate generation of vortices with predefined OAM, effectively controlling the direction and distribution of polariton flow density. Techniques employed include resonant excitation and resonant imprinting of OAM^27–29^, crafting of an effective complex trapping potential^4,5,8,12^ under incoherent excitation, mechanical rotation of the trap^30–35^, and the less understood method of directly transferring OAM from a non-resonant optical pump beam^36^. Incoherent control over polariton vortices has been demonstrated^37^ in scenarios involving short-lived polaritons with lifespans in the order of picoseconds, which precludes long-range ballistic movement. These polaritons tend to form macroscopic coherent states primarily beneath the pump spot, facilitating gain-induced trapping for such polaritons^21,38,39^.

In our study, we explore both through experimental observation and numerical simulations of the emergence of persistent polariton currents within an optically induced elliptical pot-shaped trap in a planar microcavity. Experimentally, we achieve controlled non-resonant excitation of polariton condensates, which support internal, continuous polariton flows. Interestingly, the observed two-petal configuration of the flow, against initial assumptions, does not signify the establishment of a phase-locked standing wave, as one might infer from previous studies (e.g.,^40,41^). Instead, it exists in conjunction with its phase, dynamically circulating around the trap’s center. Furthermore, we uncover the stochastic alternation between two orthogonal states of polariton currents when subjected to pulse-periodic nonresonant optical excitation, highlighting a novel aspect of polariton dynamics under such conditions.

Polariton currents that yield a nonzero OAM of the polariton condensate manifest as a twisted wavefront in its photoluminescence (PL). This distinctive feature allows for the assessment of polariton flows through the analysis of the PL phase distribution. To elucidate the circulation patterns within the polariton condensate phase, we employ interferometry measurements^4–6^. Specifically, we utilize a Mach-Zehnder interferometer equipped with a spherical reference wave, enabling precise observation and characterization of the phase dynamics associated with polariton flows.

## Results

### Experiment

**Figure 1:**
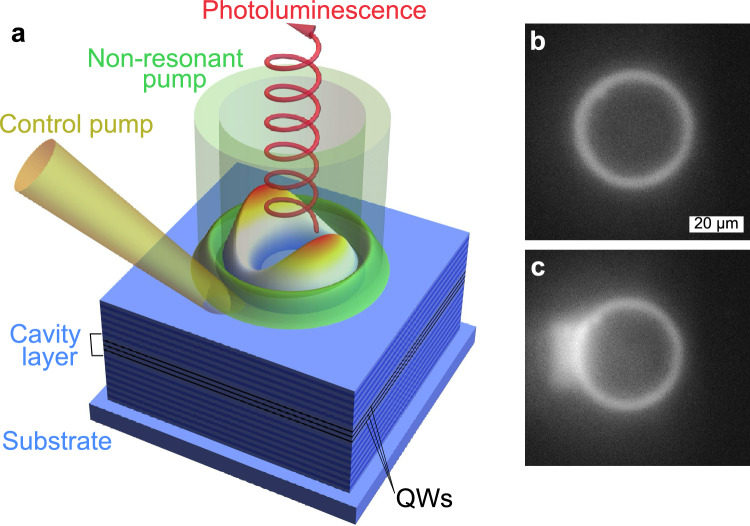
(**a**) Schematic representation of the polariton condensate excitation process using a nonresonant elliptical optical pump within a planar microcavity that contains embedded quantum wells. (**b**) Luminescence from the sample when subjected to nonresonant optical pumping at a power level below the polariton condensation threshold, without any additional control pulses. (**c**) Luminescence observed from the sample under similar conditions as in (**b**), but with the application of a supplemental control pulse (the brightness in the images has been enhanced by 50% to improve clarity).

The generation of a polariton condensate within a planar optical microcavity containing embedded quantum wells is schematically depicted in Fig. 1a. This condensate is formed through nonresonant optical pumping (targeting the upper Bragg mode of the microcavity) in a pulse-periodic regime with a pulse duration of 1 ps and an interpulse interval of 13 ns. The pump beam has the shape of an annulus that is squeezed in one direction, resulting in an ellipse rather than a perfect ring. The average radius, full width at half maximum and ellipticity of the annulus are approximately $19 \, \upmu \text {m}$, $3 \, \upmu \text {m}$ and 1.03, respectively. Further details about the sample being investigated are provided in the “Methods” section. Figure 1b illustrates the luminescence from the sample when the pump power is significantly below the threshold for polariton condensation, offering insight into the pump’s shape. Pump excites a reservoir of incoherent excitons, with a spatial distribution mirroring that of the pump beam. This exciton reservoir serves as a source of polaritons for the condensate, feeding it through stimulated relaxation processes. Concurrently, the repulsive interaction between polaritons and excitons causes the reservoir to act as a trapping potential for polaritons.

Polaritons generated within this setup have a sufficiently long lifetime (estimated to be in the tens of picoseconds), allowing them to move down the potential gradient to the trap’s bottom and occupy the trap’s eigenstates. Given the dissipative nature of polaritons and the presence of pumping, the populated eigenstate does not necessarily have to be the trap’s ground state, which in our geometry is characterized by the maximum at the center of the trap. Instead, it is the state that offers the most favorable balance between the distributed losses and gain across the microcavity plane^42–45^. This balance is influenced by the overlap between the polariton condensate wave function and the reservoir exciton cloud.

**Figure 2:**
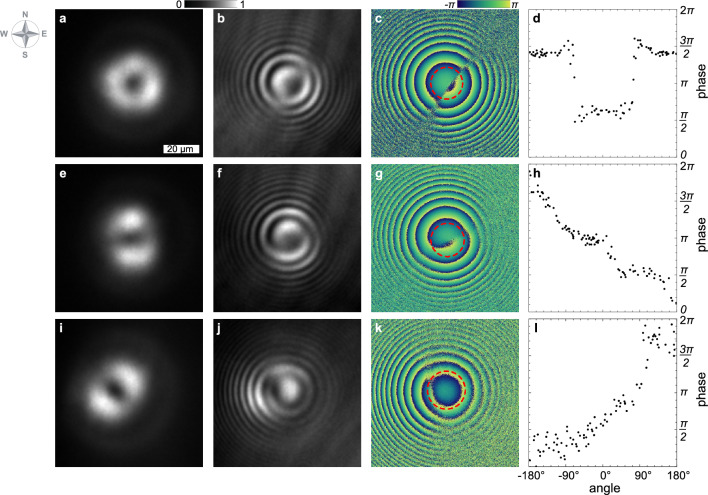
Observation of polariton condensates in an elliptical trap. From left to right: the density distribution of the polariton condensate, an interferometry image, a relative phase map of the condensate, and a depiction of phase variation along a red dashed circumference indicated in the panel to the left. (**a**–**d**) Depict the condensate during stochastic switching behavior in a pure elliptical trap. (**e**–**l**) Showcase condensates with cw and ccw polariton currents, respectively, in traps with optically imposed chirality. The angle $0 ^{\circ }$ in the right panels is arbitrarily chosen to seamlessly integrate the phase within the range from 0 to $2\pi$, avoiding any discontinuities.

**Figure 3:**
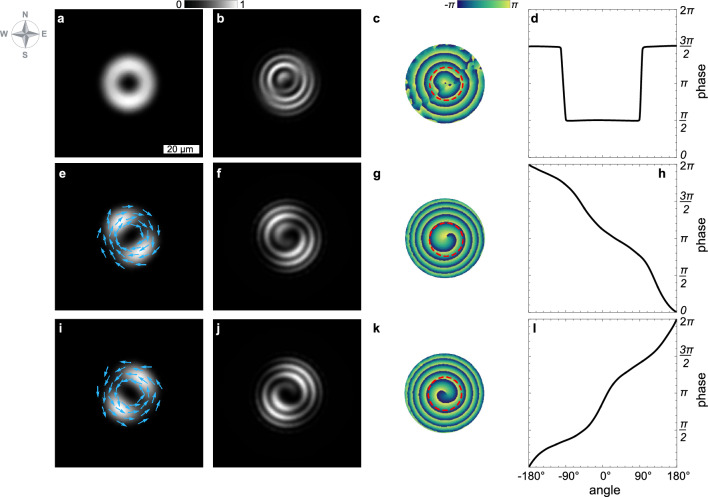
Simulation of polariton condensates within an elliptical trap is depicted, with the meaning of the panels corresponding to those described in Fig. 2. In (**e**) and (**i**), blue arrows represent the vector field of the current density $\textbf{J}$, visually illustrating the direction and magnitude of polariton flows within the condensate. The parameters employed in these simulations are detailed in the “Methods” section.

In our initial experiment, we induce the formation of a polariton condensate via optical excitation and subsequently record its time-averaged PL spatial distribution, see Fig. 2a. The resultant polariton condensate exhibits a nearly annular shape, with slight azimuthal modulation. Interference patterns between the condensate’s PL and a spherical reference wave are observed, as depicted in the interferometry image in Fig. 2b. This coherent spherical wave is produced by enlarging a segment of the condensate’s PL and passing it through a converging lens positioned further from the image plane than its focal length. The resulting interference fringes form concentric rings, bisected at two nearly opposite points, such that the semicircles of maximum intensity (white) are replaced by semicircles of minimum intensity (black). To deduce the phase of the condensate, we capture four interferometry images, denoted as $I(\textbf{r}, \Delta \phi )$, at various phase delays within the interferometer, specifically at $\Delta \phi = 0, \pi /2, \pi$ and $3 \pi /2$. From these images, the relative phase of the condensate with respect to the reference beam’s phase can be calculated as following^46^:

1 $$\begin{aligned} \Phi (\textbf{r}) + \Phi _{\text {ref}} ({r}) = \tan ^{-1} \left[ \frac{ I (\textbf{r}, 3 \pi /2) - I (\textbf{r}, \pi /2) }{ I (\textbf{r}, 0) - I (\textbf{r},\pi )} \right] . \end{aligned}$$

The extracted phase distribution is depicted in Fig. 2c, with an additional illustration of the phase variation around the trap’s center along the condensate’s ridge, presented in Fig. 2d. It is noticeable that the extracted phase undergoes two jumps (by $-\pi$ and $+\pi$) when encircling the trap in a closed circular loop, while remaining constant outside of these jumps. This characteristic suggests the possibility of dips in the density distribution of the condensate around the areas of phase jumps. However, such an expectation does not align with the observations shown in Fig. 2a, where the density appears almost homogeneous in the azimuthal direction. This inconsistency leads to the conclusion that the PL we observe represents an average over multiple realizations of condensates with arbitrary phases, rather than depicting a single condensate with a specific phase distribution.

The elliptical trap maintains axial symmetry, ensuring that the counterclockwise (ccw) and clockwise (cw) directions are equivalent. Consequently, this symmetry leads to an equal probability of observing ccw and cw polariton currents within the condensate following each pump pulse. Averaging across multiple pulses results in an interferometry image that aggregates phase characteristics unique to each realization of the condensate observed over the duration of the experiment.

To differentiate between the polariton current states contributing to PL observed in Fig. 2a, we introduce chirality to the system, disrupting the equivalence of the ccw and cw directions. We achieve this by augmenting each elliptical pump pulse with a weak control pulse shaped as a stripe that overlaps with the ellipse. The position of this stripe can be varied at will, altering the shape of the effective optically induced potential. Figure 1c displays the sub-threshold PL from the sample when subjected to the additional control pulse, illustrating the modification in the potential landscape induced by this method.

The middle (panels e–h) and lower (panels i–l) rows of Fig. 2 depict two polariton condensates created with control pulses shifted to the southwest and northwest of the center of the trap, respectively. Each condensate displays a dumbbell shape, with their symmetry axes oriented at angles to one another. A distinguishing feature of these states, setting them apart from the conventional first excited states in a 2D pot-shaped (including harmonic) trap, is their vortical nature, characterized by internal circular polariton currents. This inference is drawn from the interference patterns of PL with a spherical wave, as observed in Fig. 2f and j, where distinct single-armed spirals reveal the existence of vortices with topological charges of $m=-1$ and $+1$, respectively. The phase distributions shown in Fig. 2g–h and k–l, which demonstrate a phase shift of $-2\pi$ and $+2\pi$ in a complete circuit around the trap center, respectively, further substantiate our findings. We consider these vortex polariton condensate states as significant contributors to the PL and the interferometry image presented in panels a and b of Fig. 2.

### Simulations

To substantiate our observations, we complement our experiment with numerical simulations of the polariton condensate’s behavior under the optically induced annular pump. The details of the numerical model are given in the “Methods” section. The results of our simulations, as depicted in Fig. 3, successfully qualitatively mimic the experimental findings shown in Fig. 2. In Fig. 3a and b, we present the density distribution and interference image, averaged across multiple distinct realizations of the polariton condensate, featuring vortices with opposite circulation directions. The phase distribution and azimuthal phase variation displayed in Fig. 3c and d are derived from Fig. 3b employing the extended Fourier-transform method for analyzing closed-fringe patterns, see^4^. Our simulations capture both the displacement of interference fringes and the compensating phase jumps amidst a relatively uniform azimuthal density distribution. It is noteworthy that increasing the number of realizations for averaging, from two to any larger arbitrary number, does not alter the observed patterns.

The middle (panels e–h) and lower (panels i–l) rows in Fig. 3 showcase the vortex states of polariton condensates, that contribute to the stochastic switching observed in panels a–d. These simulations replicate the emergence of dumbbell-shaped condensates with swirling phases that vary monotonically around the core of the vortex. To reproduce these states, we supplemented the elliptical pump with a control beam, the shapes of which was designed to mimic that depicted in Fig. 1c. Details of the supplemental beam are given in the “Methods” section. For Fig. 3e–h and i–l, the control beams are shifted to the southwest and northwest of the center of the trap by ${\mp }4.5\,\upmu \text {m}$, respectively. The polariton currents, represented by the vector field of the current density $\textbf{J} = \text {Im} \left( \Psi ^* \nabla \Psi \right)$, are indicated by blue arrows in panels 3e and i, further elucidating the dynamics within these condensate states.

## Discussion

In this study, we have demonstrated a regime of stochastic switching of polariton condensate vortices (circular currents) within a complex elliptical trapping potential. Through PL measurements, we observed that the time-averaged intensity distribution forms a ring shape with slight azimuthal modulation. Interferometry measurements, conducted with a spherical reference wave, revealed that the averaged interference fringes consist of concentric rings bisected at points located on opposite sides of the condensate’s symmetry axis. By introducing a supplemental weak control pump pulse, we were able to induce chirality in the trapping potential. This modification shifts the system into a regime of deterministic circulation. In this regime, the emergence of polariton vortices and the direction of their circulation are influenced by the positioning of the control pulse. This finding highlights the control and manipulation possibilities of polariton currents in optically induced potentials.

It is important to clarify that, within the stochastic switching regime, as observed in Figs. 2b and 3b, the process involves averaging over intensity, meaning that the extracted phase distributions in Figs. 2c and 3c do not reflect the phase characteristics of any specific contributing condensate. Nevertheless, interferometry measurements enable a clear distinction between the stochastic and deterministic regimes in the behavior of the polariton condensate, without the need for comprehensive analysis. This distinct advantage sets the interferometry method apart from other techniques used to uncover the phase properties of polariton condensates, such as the OAM sorting method^47,48^ and the machine learning approach that employs dimensionality reduction and linear regression techniques^49^.

In our experiments focused on the deterministic circulation of polaritons, the annular pump pulse and the weak control pulse are directed at the sample to arrive almost simultaneously. Nonetheless, it is common to encounter a delay between these pulses. We have observed that the creation of circular polariton currents in a predetermined direction remains unaffected regardless of which pulse arrives first, at least within a delay interval of $\pm 500$ ps. This finding leads us to infer that the lifetime of the optically induced reservoir excitons, which contribute to the trapping potential, spans hundreds of picoseconds.

In all our experiments, PL from the condensate region was detected continuously while the optical pump was active. Once initiated, both the PL and interferometry images remained stable until the pump was either deactivated or its operational regime was modified. Notably, in the stochastic switching regime, the locations where the interference fringes broke were consistent across different experimental runs, even after the pump was turned off and then back on. This consistency can be attributed to the fringes being dictated by the phase difference between the condensate’s PL and the reference wave. This phase difference is influenced by the location of the condensate spot used to generate the reference wave and by the phase delay between the interferometer’s arms, which remain constant throughout the experiment. The unchanging nature of the reference wave’s phase and the interferometer’s delay also explains why the averaged interferometry images exhibit clear interference fringes instead of a uniformly illuminated spot. This indicates that the phase difference with the reference wave for polariton condensate vortices, regardless of their circulation direction, is maintained. The stochastic behavior observed could stem from either classical or quantum phenomena. Our current experimental setup does not allow us to definitively favor one explanation over the other, leaving the door open for further investigation into the underlying nature of this stochasticity.

## Methods

### Sample details

The sample under study is a planar $5 \lambda /2$ AlGaAs microcavity with an embedded set of GaAs QWs, sandwiched between two distributed Bragg reflectors, formed of 31 (top) and 35 (bottom) AlGaAs/AlAs pairs of layers. The bottom mirror is grown on the GaAs substrate. The cavity quality factor is estimated as $Q > 10^{4}$. Across the sample, the detuning between the QW exciton resonance and the cavity mode spans from $-20$ meV to $+3$ meV. The strong exciton-photon coupling regime is supported by the sample with the Rabi splitting of 9.2 meV.

### Experimental setup

The experimental setup is designed to provide precise control and detailed analysis of the polariton condensates formed within the sample. The sample itself is cooled to approximately 6 K using a low-vibration closed-cycle exchange-gas cryostat, ensuring minimal thermal fluctuations during the experiments. For optical pumping, a ps-pulsed Ti:Sapphire laser is employed, tuned to the second dip of the reflectance spectrum on the high-energy side of the distributed DBR at 751 nm. This laser beam is split into two separate beams with an intensity ratio of 8:92. The beam with higher power passes through an acousto-optic modulator for intensity adjustment and a MEMS-based spatial light modulator, creating a ring-shaped trapping pulse. The beam with lower power is directed through a 300 mm mechanical delay stage and a slit-based spatial filter, forming a control pulse of small intensity, which can be toggled by a mechanical shutter. Both trapping and control beams are focused onto the sample into a spot approximately 20 μm in diameter using a microscope objective with a focal length of 4 mm. The objective’s relatively high numerical aperture (${NA }=0.42$) facilitates both real-space and reciprocal-space analysis of the trapped polariton condensates. Photoluminescence emitted from the condensates is collected in reflection geometry using the same objective. This PL is distinguished from the back-reflected laser light by a long-pass interference filter and then split into two pathways. One pathway directs the first PL beam through the back focal plane of the objective into a slit of a 0.5 m imaging spectrometer, allowing for reciprocal space analysis of the condensate emission. The second pathway involves analysis in real space, utilizing a Mach-Zehnder interferometer where one arm contains a lens with a focal length of $F=60$ mm, thereby using a segment of the condensate’s glow as a reference spherical wave. The object arm of the interferometer is adjustable via a precision mechanical delay line to align optical paths accurately within the decay range of the first-order correlation function. The interference pattern is magnified by a factor of 62.5 and captured by a thermoelectrically cooled CMOS camera with exposure times ranging from 0.5 to 2.5 s. Multiple interferograms are recorded with slight adjustments to the interferometer’s path difference, capturing images at phase delays of $\Delta \phi = 0$, $\pi /2$, $\pi$, and $3\pi /2$. The measured interferogram gives

2 $$\begin{aligned} I(\textbf{r},\Delta \phi ) = I'(\textbf{r}) + I''(\textbf{r})\cdot \cos [\Theta (\textbf{r})+\Delta \phi ]. \end{aligned}$$

Here, $I'(\textbf{r}) = I_\mathrm{sig} + I_\mathrm{ref}$, $I''(\textbf{r}) = 2\sqrt{ I_\mathrm{sig} I_\mathrm{ref}}$, where $I_\mathrm{sig}$ and $I_\mathrm{ref}$ are the intensities of the object and reference waves. The relative phase difference, $\Theta (\textbf{r})$, can be recovered using a four-step phase-reconstruction algorithm^50^ as follows:

3 $$\begin{aligned} \Theta (\textbf{r}) = \tan ^{-1} \left[ \frac{I(\textbf{r}, 3\pi /2) - I(\textbf{r},\pi /2)}{I(\textbf{r}, 0) - I(\textbf{r},\pi )}\right] . \end{aligned}$$

The rest parts of the interferogram can be obtained accordingly:

4a $$\begin{aligned}{}&I'(\textbf{r}) = [I(\textbf{r},0) + I(\textbf{r},\pi )]/2, \end{aligned}$$

4b $$\begin{aligned}{}&I''(\textbf{r})\cdot \sin \Theta (\textbf{r}) = [I(\textbf{r},3\pi /2) - I(\textbf{r},\pi /2)]/2. \end{aligned}$$

For a more comprehensive analysis of the data, a separate measurement was conducted where the object arm of the interferometer was obstructed by a mechanical shutter. This setup was used to exclusively gather the intensity data of the reference wave, denoted as $I_\mathrm{ref}$. It is assumed for this measurement that the reference beam maintains a uniform intensity across the entire sensor area. Moreover, the phase of the reference beam, $\Phi _\mathrm{ref}(\textbf{r})$, is characterized by a slowly varying parabolic shape, with its vertex centered at the origin of the object wave’s coordinate system. In this case, the complex field of the condensate luminescence, $\Psi (\textbf{r}) = \Vert \Psi (\textbf{r})\Vert \cdot \exp [i\,\Phi (\textbf{r})]$, can be evaluated as follows^5^

5a $$\begin{aligned}{}&\Vert \Psi (\textbf{r})\Vert = I'(\textbf{r}) - I_\mathrm{ref}, \end{aligned}$$

5b $$\begin{aligned}{}&\Phi (\textbf{r}) = \Theta (\textbf{r}) - \Phi _\mathrm{ref}(\textbf{r}). \end{aligned}$$

This approach does not require the complex 2D Fourier analysis typically used in off-axis digital holography experiments.

### The model used for numerical simulations

To describe the evolution of the polariton condensate, the generalized Gross-Pitaevskii equation is employed for the polariton wave function $\Psi (t,\textbf{r})$^51,52^:

6 $$\begin{aligned} i \hbar \partial _t \Psi (t,\textbf{r}) = \left\{ [i \Lambda _0 n_{\text {R}} (t,\textbf{r}) - 1] \frac{\hbar^2 k^2}{2 M} \nabla ^2 + \alpha |\Psi (t,\textbf{r})|^2 + \alpha _{\text {R}} n_{\text {R}} (t,\textbf{r}) + \frac{i \hbar}{2} \left[ \rho n_{\text {R}} (t,\textbf{r}) - \gamma \right] \right\} \Psi (t,\textbf{r}), \end{aligned}$$

coupled to the rate equation for the density of the exciton reservoir $n_{\text {R}} (t,\textbf{r})$:

7 $$\begin{aligned} \partial _t n_{\text {R}} (t,\textbf{r}) = P (\textbf{r}) - \left[ \gamma _{\text {R}} + \rho |\Psi (t,\textbf{r})|^2 \right] n_{\text {R}} (t,\textbf{r}). \end{aligned}$$

In Eq. (6), *M* is the effective mass of polaritons in the microcavity plane, $\alpha$ and $\alpha _{\text {R}}$ are interaction constants of polaritons with each other within the condensate and polaritons with the reservoir excitons, respectively. The rightmost square brackets in Eq. (6) are responsible for the balance of gain and losses in the polariton condensate. *ρ* is the stimulated scattering rate from the reservoir to the condensate, $\gamma$ and $\gamma _{\text {R}}$ are the decay rates of polaritons and reservoir excitons. The reservoir is excited by the optical pump $P(\textbf{r}) = P_{1} (\textbf{r}) + P_{2} (\textbf{r})$ composed of two components. The first component is responsible for the excitation of the elliptical trap $P_1(\textbf{r}) \propto \exp \left[ -({r}_1 - R )^2 /2 w^2 \right]$, with ${r}_1 = \sqrt{x^2 + (y/s)^2}$, where *R*, *w* and *s* are the average radius, width and ellipticity of the trap. The second component $P_2(\textbf{r}) \propto p_{2}\exp \left\{ -[(x-d_x)/w_x]^4 -[(y-d_y)/w_y]^4\right\}$ is responsible for the supplemental control beam, which imposes chirality on the system. $d_{x,y}$ and $w_{x,y}$ are the shifts and widths of the beam in the corresponding directions, respectively. $p_2$ characterizes the power ratio of the second component relative to the power of the first component.

In Eq. (6), the imaginary part of the kinetic energy term incorporates the energy relaxation of propagating polaritons, which arises from their interaction with reservoir excitons^51,52^. The extent of the relaxation is heavily dependent on the overlap between the condensate wave function and the reservoir. $\Lambda _0$ serves as a fitting parameter within this context, quantifying the influence of energy relaxation on the polaritons.

### Values of the parameters

We take the following values of the parameters for numerical simulations. The effective mass of polaritons is $M = 4 \cdot 10^{-5} m_{\text {e}}$, where $m_{\text {e}}$ is the free electron mass. The polariton and exciton decay rates are taken as $\gamma = 0.025$ ps^-1^ and $\gamma _{\text {R}} = 0.01$ ps^-1^, respectively. The stimulated scattering rate is taken as $\hbar \rho=0.1\, \text {meV} \, \upmu \text {m}^2$. The nonlinearity coefficients are taken as $\alpha = \alpha _{\text {R}}/2 = 3 \, \upmu \text {eV} \, \mu \text {m}^2$. The average radius, width and ellipticity of the pump are $R = 19 \, \upmu \text {m}$, $w = 2 \, \upmu \text {m}$ (corresponding to full width at half maximum approximately $4.7\, \upmu \text {m}$) and $s=1.03$, respectively. The width *w* was chosen larger than in experiment to account for the diffusion of the reservoir excitons away from the pump spot, see the Supplemental Material for Ref.^53^. The parameters characterizing the supplemental pump component are following: $w_x = 6 \, \upmu \text {m}$, $w_y = 8 \, \upmu \text {m}$, $d_x = - 9 \, \upmu \text {m}$ and $p_2 = 0.025$.

## Data Availability

All data generated and analysed during the current study are available from the corresponding author on reasonable request.
